# High *I*_on_/*I*_off_ current ratio graphene field effect transistor: the role of line defect

**DOI:** 10.3762/bjnano.6.210

**Published:** 2015-10-23

**Authors:** Mohammad Hadi Tajarrod, Hassan Rasooli Saghai

**Affiliations:** 1 Department of Electrical Engineering, Tabriz Branch, Islamic Azad University, Tabriz, Iran

**Keywords:** field effect transistor, graphene, line defect

## Abstract

The present paper casts light upon the performance of an armchair graphene nanoribbon (AGNR) field effect transistor in the presence of one-dimensional topological defects. The defects containing 5–8–5 sp^2^-hybridized carbon rings were placed in a perfect graphene sheet. The atomic scale behavior of the transistor was investigated in the non-equilibrium Green's function (NEGF) and tight-binding Hamiltonian frameworks. AGNRFET basic terms such as the on/off current, transconductance and subthreshold swing were investigated along with the extended line defect (ELD). The results indicated that the presence of ELDs had a significant effect on the parameters of the GNRFET. Compared to conventional transistors, the increase of the *I*_on_/*I*_off_ ratio in graphene transistors with ELDs enhances their applicability in digital devices.

## Introduction

Graphene, a two-dimensional allotrope of carbon with the thickness of one atom, has attracted the attention of researchers because of its unique electronic transport properties. The properties of graphene such ultra-thin body properties for optimum electrostatic scaling and excellent thermal conductivity has made it a potential alternative to silicon and facilitated the manufacture of devices [1–2]. Furthermore, the high carrier mobility and velocity of graphene is utilized in ballistic and high switching speeds devices [3–4]. However, the very large off-current of graphene at room temperature, which is associated with the small band gap, renders it incapable of being integrated as a building block for pure carbon-based transistor devices [5–6]. The electronic properties of graphene are the result of its particular structure. In order to modify the transport behavior, the physical structure of graphene needs to be changed. Consequently, topological defects such as vacancies, impurities, adatoms and Stone–Wales defects are the best candidates for changing the hexagon structure of graphene with acceptable C–C distances and angles for sp^2^ hybridization [7]. These defects play a remarkable role in graphene and nano-structured devices. One controlled defect in graphene are grain boundaries. The electrical and thermal conductivity decrease with grain boundaries in materials [8–9]. By studying the grain boundaries in graphite, extended line defects become visible in the STM analysis [10]. The first experimental report of the extended line defect (ELD), which was studied through alternating Stone–Thrower–Wales defects, was presented by Lahiri et al. [11]. Applying defects and divacancies in a pristine sheet creates a 5–8–5 line defect. They found that one-dimensionally extended defects could be used as metallic wire. Following this study, a valley filter, based on line defects scattering in graphene, was introduced by Gunlycke et al. [12]. Later, by applying the tight-binding calculation, Bahamon et al. demonstrated metallic characteristics and Fabry–Pérot oscillation phenomena in graphene [13]. Afterwards, some works addressed the effect of the extended line defect along the edges. For example, G. P. Tang et al. showed that spin polarization and carrier transport in zigzag graphene nanoribbons (ZGNR) were mainly dependent on the position of the line defect [14]. In addition, Hu et al. investigated the electrical and magnetic properties of zigzag edge graphene under external strain. They found that the local magnetic moments on the line defect were amplified by strain, coupling this with the edge magnetic moments caused a modification of the spin polarization on one edge [15]. Some studies investigating the effect of line defects on zigzag nanoribbon could have been motivated by the fact that the line defect develops along the edge of zigzag nanoribbons. There are also studies on the line defects in armchair nanoribbons [16–17]. The research into divacancies and ELD in armchair nanoribbons shows that the presence of divacancy defects has signiﬁcant impacts on the band structure and electronic transport properties of AGNR. However, no study has yet been conducted on the effect of the extended line defect on field effect transistors. In the present study, first, the device performance of an AGNR field effect transistor with ELD was investigated by employing self-consistent NEGF formalism and tight-binding Hamiltonian calculation. Then various parameters of the transistor, e.g., on/off current, transconductance and subthreshold swing, were calculated, and the influence of the line defect in different channel sizes was compared to the ideal AGNRFET.

## Model

**Figure 1:**
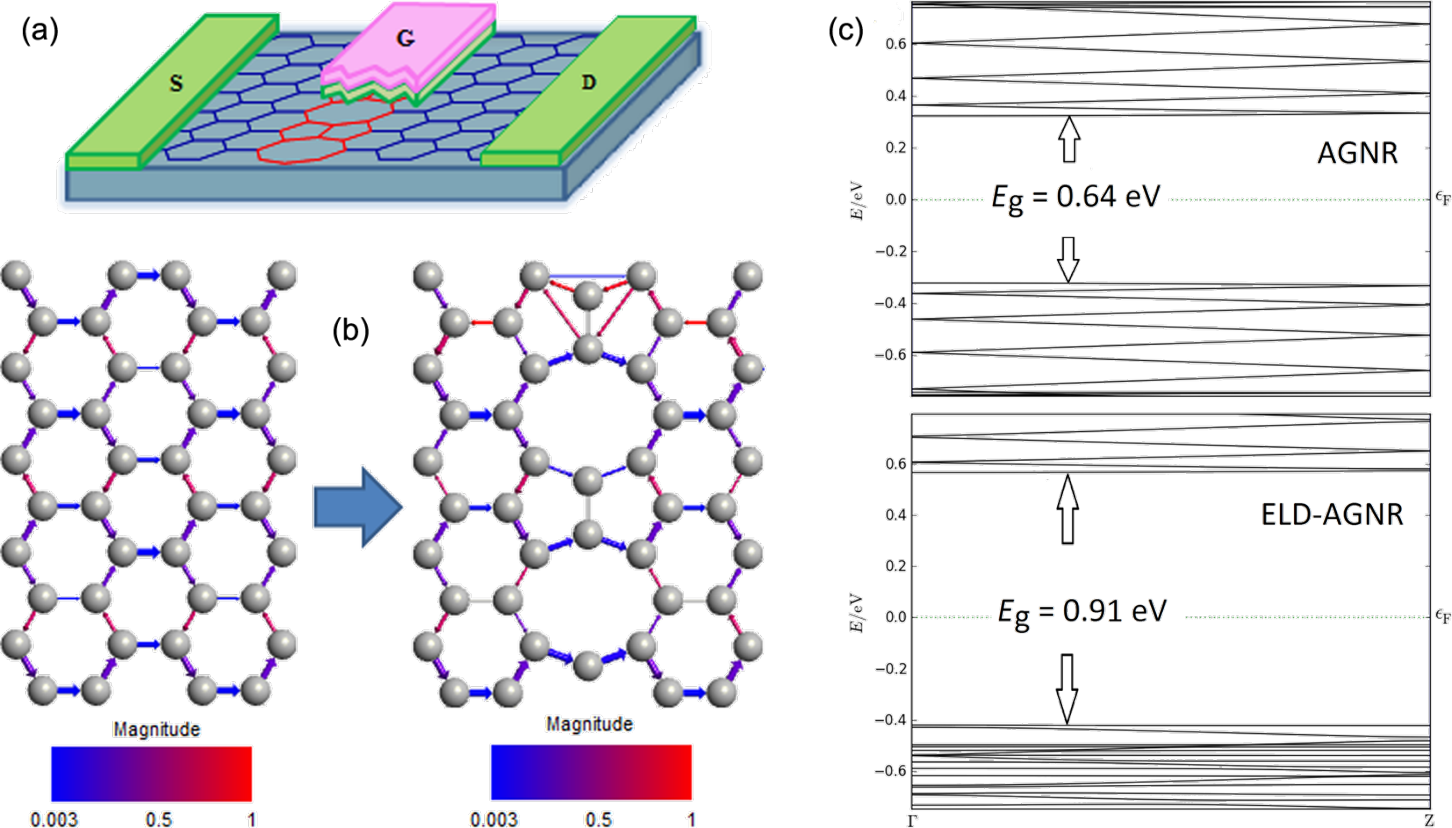
(a) Schematic sketch of a simulated GNRFET with an extended line defect in the center of the channel, N-type contacts, Al_2_O_3_ gate dielectric and SiO_2_ substrate. (b) Transmission pathway along 1.8 × 20 nm armchair graphene nanoribbon. The arrows show the magnitude of carrier transport. (c) Band structure of 1.8 × 20 nm AGNR (top image) and ELD-AGNR (bottom image).

Figure 1a illustrates the schematic diagram of GNRFET channel, which contains a 5–8–5 extended line defect in the center of the armchair graphene nanoribbon. The C–C bond lengths in the line defect are between 1.43–1.83 Å, that indicating sp^2^ hybridization [18]. The tight-binding model with the first nearest-neighbor approximation was used to model the electrical structure of the transistor channel, which conformed to the first-principles calculations used to describe the electronic band structure of ELD-AGNRs [19–20]. The Hamiltonian computation in this system was separated into AGNR (H_A_), line defect (H_D_) and coupling between AGNR and the defect (H_C_). The relevant formulas are as follows [21]:

[1]
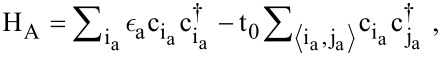


[2]
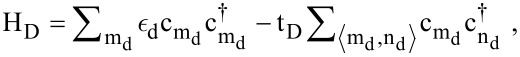


[3]
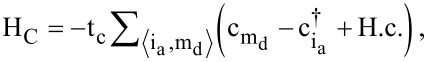


where 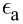 and 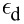 are the on-site energies in the AGNR and the line defect, respectively; t_0_ (t_D_) is the hopping energy of the AGNR (line defect); t_c_ denotes the coupling between AGNR and line defect; H.c. stands for the Hermitian conjugate; indices i_a_ and m_d_ are the site coordinates in the AGNR and line defect respectively; <i_a_,j_a_>(<m_d_,n_d_>) represent the pair of nearest neighbors. Thus, the whole Hamiltonian system is defined as a three-part summation (H = H_A_ + H_D_ + H_C_). Non-equilibrium Green's functions method was employed to simulate the device performance in the atomic scale simulation. In the general context of nano-transistor modeling, the retarded Green's function of the channel in between the right and left contacts, which can be evaluated as follows [22]:

[4]
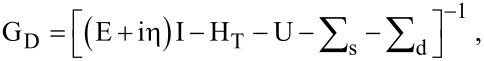


where H_T_ is the tight-binding Hamiltonian matrix of the GNR channel, which can be evaluated using a tight-binding model as explained before; 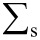 and 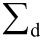 are the self-energies describing the coupling between the device and source and drain region, respectively; U is the self-consistent potential matrix determined by the solution of a 3D Poisson equation; and η is an inﬁnitesimally small quantity. Accordingly, the transmission probability of carriers through the device can be written as:

[5]
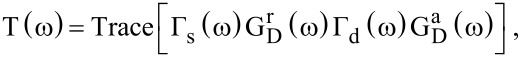


where 
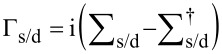
 is the coupling between source (drain) contact and the device region; 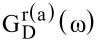 represents the retarded (advanced) Green's function for the device region, which follows the relationship of G^a^ = [G^r^]^†^, 
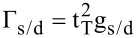
 with g_s/d_ being the surface-state Green’s functions of the source and drain contacts. Here, g_s/d_ are solved by sticking out a semi-infinite one dimensional double-atom chain from semi-infinite AGNR in the Green’s function space [23]. The Green’s function coefficients extracted by derivation, 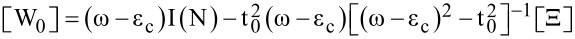, 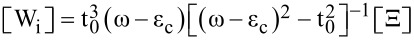 and 
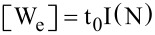
, are the on-site energy, the coupling between the two atoms in each primitive cell, and the coupling between the neighboring two primitive cells of the chain, respectively. For AGNR with even width (M), N = M/2 and [Ξ] = 2δ_jl_ – δ_11_ + δ_j,l+1_ + δ_j,l−1_. Otherwise, N = (M – 1)/2 and [Ξ] = δ_jl_ + δ_j,l+1_ + δ_j,l−1_. To transform the double-atom chain into its molecular orbit representation, diagonalization of the matrix [Ξ] is performed, allowing the surface-states Green’s function of the semi-infinite AGNR to be found. The current can be calculated by using the Landauer formula [24]:

[6]
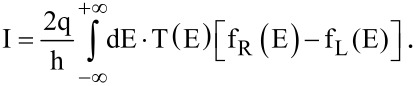


Here, f_R_ and f_L_ are the Fermi functions of the right and the left contact, respectively.

## Results and Discussion

In this section, the carrier transport and electrical properties of ELD-GNRFET are reported. Figure 1a is the schematic diagram of the transistor. The transistor device contains a top gate structure of Al_2_O_3_ (ε_r_ = 9.8) gate oxide embedded along the gate and a channel with 2 nm width. The armchair nanoribbon channel used with the extended line defect was placed in the center. The drain and source contacts were doped with concentrations of 1 × 10^9^ m^−1^. Room temperature (300 K) operation was assumed in all simulations and the bias voltage was set to *V*_d_ = 0.7 V. Depending on the edge geometry, ELDs take different configurations. The same configuration of line defects was assumed in all simulations [25] (see in Figure 1b).

Figure 1b shows transport pathways in an ideal GNR nanoribbon and in one with an ELD. The addition of the line defect reduces the paths of conducting channels, making larger effective transport gaps. However, with the reduction of paths, the mobility and carrier density at higher energies is extremely reduced. Figure 1c shows the band structure of ideal and defect AGNR. Compared to the ideal AGNR, the band gap of ELD-AGNR is increased up to 0.91 eV (assuming the line defect in the configuration), owing to the reduction of conducting channels in ELD-AGNR.

**Figure 2:**
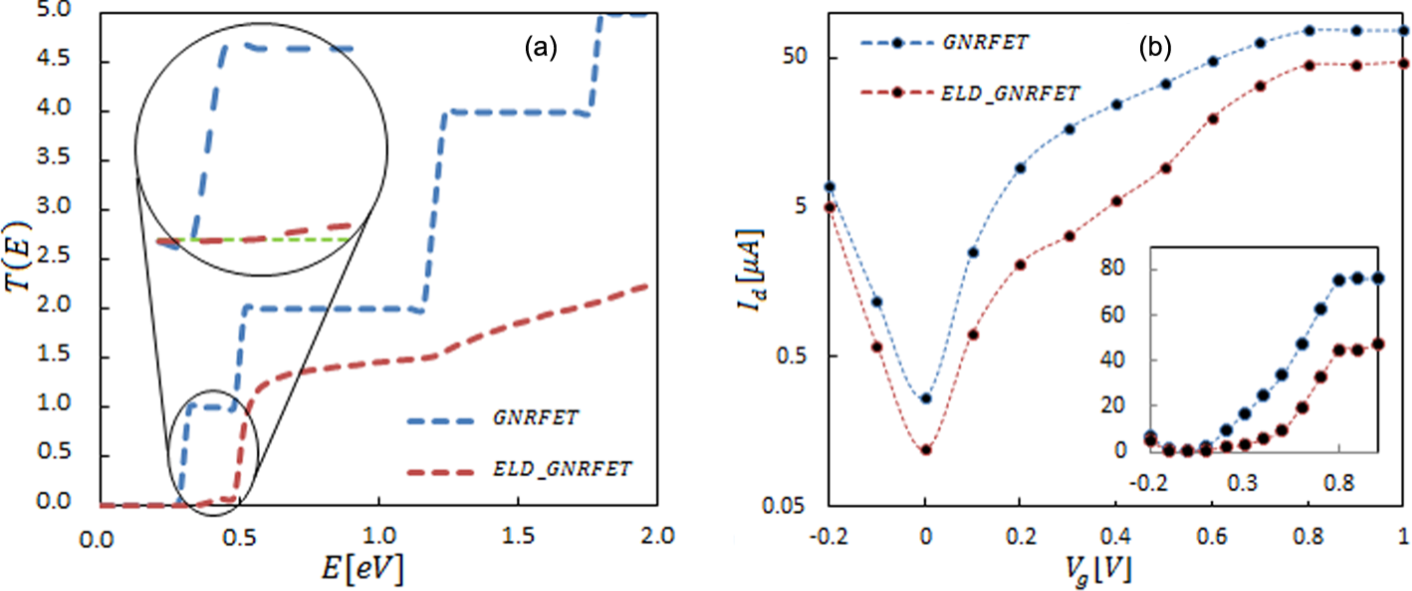
(a) Comparison between average transmission probabilities as a function of the energy in 1.8 × 20 nm armchair graphene nanoribbons. Inset shows transmission probabilities compared to the minimum effective band structure transmission (dashed green). (b) *I*_d_ vs *V*_g_ characteristics (logarithmic scale) for ELD-GNRFET (dashed red) and GNRFET (dashed blue) containing a 1.8 × 20 nm armchair graphene nanoribbon channel. The inset shows linearly scaled transfer characteristics. (*I*_on_ extracted from *I*_ds_ at *V*_gs_ = *V*_off_ + *V*_DD_ (*V*_DD_ = 0.7 V), where *V*_off_ = *V*_gs_ at *I*_ds_ = *I*_off_). Symbols show the exact value, and dashed lines are fitted to data points.

The transmission probability in Figure 2a clearly shows localized energy states in these two devices. To describe the channel performance, a probability of transmission below 10^−2^, which has no experimentally signiﬁcance, is regarded as the reference for the transmission of the defined bulk transport gap [26–27]. The result indicates that the transmission spectrum is influenced by adding a line defect. The reduction of the transmission that follows the widening of the effective band gap (Figure 1c) is considerable. Figure 2b shows the transfer characteristics of the transistors. As shown in the figure, compared to the ideal GNRFET, ELD-GNRFET has a lower current value in various gate voltages. Thanks to a significant increase of the effective band gap, off-current decrease is much more significant than the on-current reduction in ELD-GNRFET. The results indicate that on/off ratio improved 30% in the defected transistor. The simulating of the average of transconductance and the subthreshold swing describe the switching-on and the immunity of the short channel effects in the switching-off performance. In this study, the transconductance decreased from *g*_m_ = 94 μS in the ideal transistor to *g*_m_ = 55.7 μS for the defected transistor. The subthreshold swing, which is defined as ∆*V*_G_/∆log(∆*I*_D_) at the subthreshold region in GNRFET, was *S* = 166.9 mV/dec, whereas in ELD-GNRFET, reduction in the tunneling current made a higher subthreshold swing (*S* = 209.7 mV/dec) [28].

### Length effect

**Figure 3:**
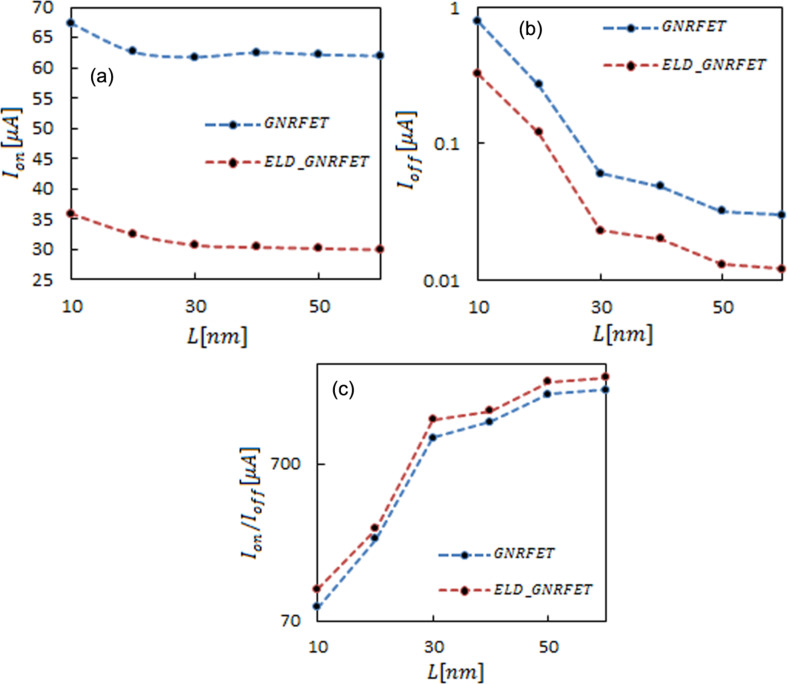
Channel length dependence of (a) on-current (b) off current (c) on/off current. The lines are a guide to the eye. The dashed blue line is for ELD-GNRFET and the dashed red line is for GNRFET.

On-current and off-current values for channel lengths from 10 to 60 nm are shown in Figure 3a and Figure 3b. Because of the decrease of the tunneling current in longer channels, the off-current decreases when the channel length is increased. Due to the increase of the band gap in the defected channels, the off-current in ELD-GNRFETs (about 70%) was smaller than that in ideal transistors. The transmission reduction in ELD-GNRFET caused an on-current decrease by 47%. Nevertheless, as seen in Figure 3c, the on/off current increased by a factor of 1.30 (30% improvement).

**Figure 4:**
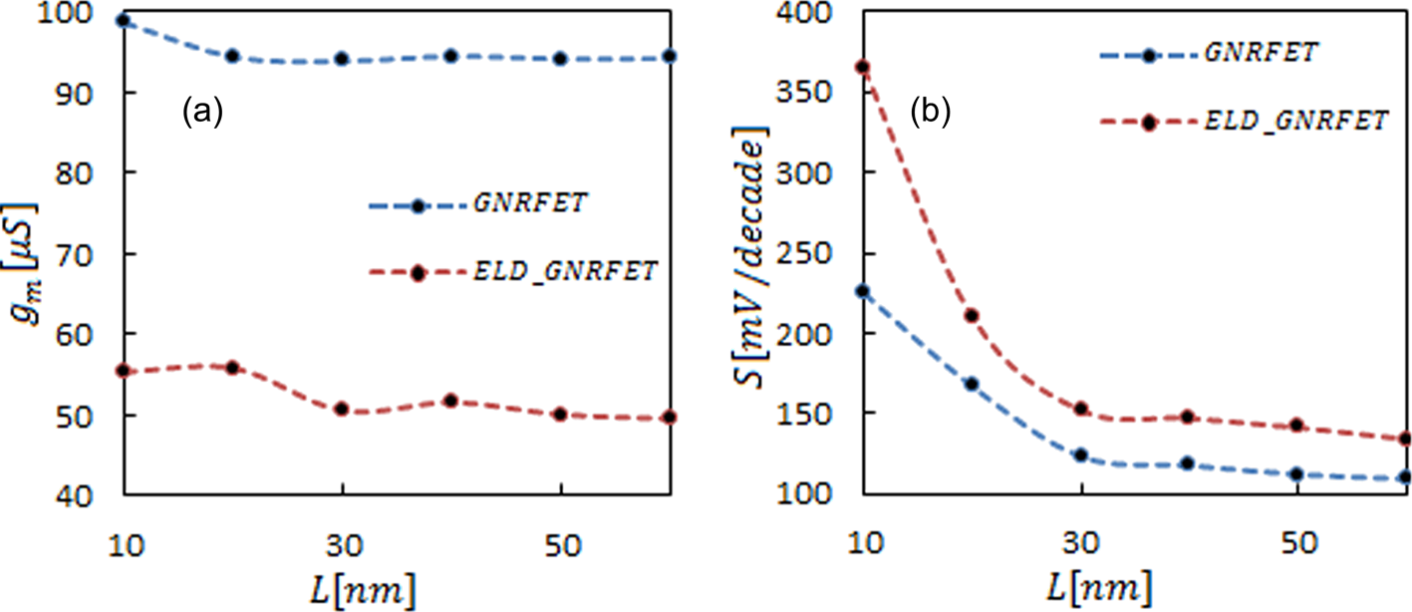
Comparison between average of (a) transconductance and (b) subthreshold swing of ELD-GNRFET and GNRFET as a function of the channel length. The lines are a guide to the eye.

Due to ballistic transport, the transconductance remains constant when the channel length is reduced. Although in the defect channel transconductance is reduced because of a smaller transition probability. Figure 4a shows that by introducing an ELD in the transistors, the transconductance decreases in various channels. In Figure 4b, the subthreshold swings as a function of the device length are compared in the defect and the ideal transistor. Stronger electrostatic control of the gate at larger lengths leads to a decrease of the subthreshold swing in both devices. However, in the short-channel devices a decrease of the tunneling current in ELD-GNRFETs result in a smaller subthreshold swing.

### Width effect

**Figure 5:**
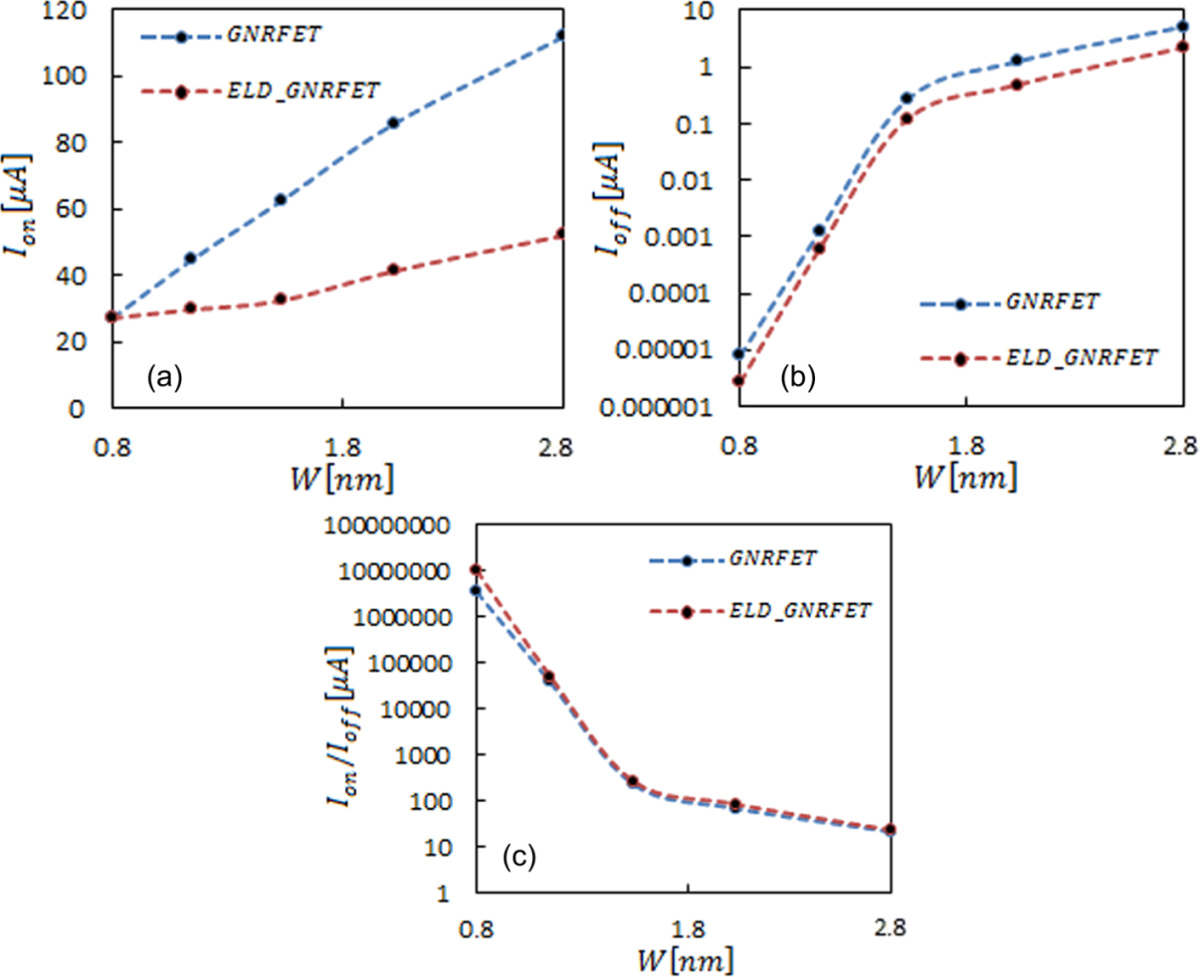
Channel width dependence of (a) on-current (b) off current (c) on/off current. The lines are a guide to the eye. The dashed blue line is for ELD-GNRFET and the dashed red line is for GNRFET.

Studies on the width of graphene show that GNR nanoribbon band gap decreases for larger nanoribbons. Additionally, the localization of carriers in GNR nanoribbon with the line defect contributes to the increase of the band gap. Figure 5a and Figure 5b show on-current and off current versus channel width (0.80–2.77 nm). The increase of the channel led to the increase of the on-current, and the increase of the off-current caused the band gap at large widths to decrease even more. The proportion of the conducting channels in defect vs ideal graphene nanoribbons increased by increasing the width of the nanoribbon, and, accordingly, the slope of on-current became gentler in the defected device (Figure 5c).

**Figure 6:**
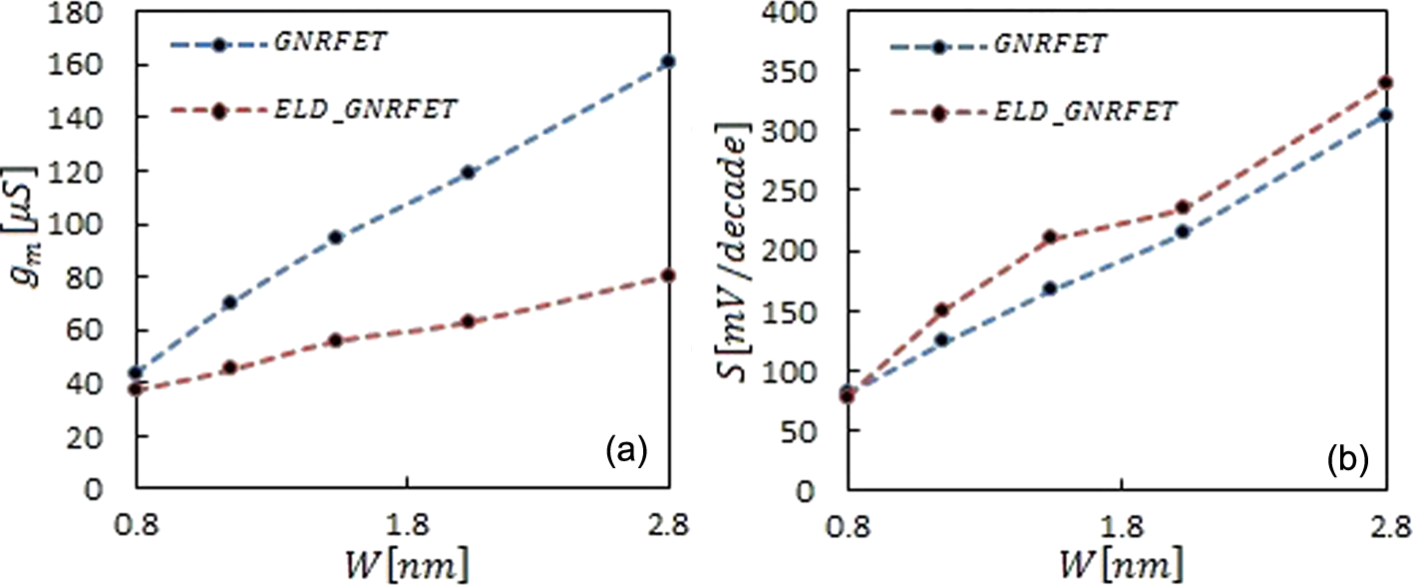
Comparison between average of (a) transconductance and (b) subthreshold swing of ELD-GNRFET and GNRFET as a function of the channel width. Symbols show the exact value, and dashed lines are fitted to data points.

Because of the reduction of the tunneling current, the off-current in ELD-GNRFET transistors decreases by factor of ca. 2.5 compared to GNRFET transistors. Overall, the on/off current ratio increased in various widths with the line defect attendance. The overall increase in terms of transconductance and subthreshold swing in Figure 6 related to the increase in conducting channel and decrease in the band gap, respectively. Despite this, in the defected device, the decreased transmission probability caused light growth in the transconductance, while less tunneling current increase the subthreshold swing.

## Conclusion

In this study a comprehensive numerical analysis was conducted about graphene nanoribbon field effect transistors with extended line defects (ELD-GNRFET) based on the NEGF formalism. According to the simulation results, applying a perpendicular line defect in an AGNR channel led to the decrease of the transport paths, causing a larger band gap in the device. The transistor behavior examined in different geometries showed that on/off current and transconductance decreased and subthreshold swing increased by adding line defect. However, the *I*_on_/*I*_off_ ratio recognized as a considerable problem in graphene transistors, increased in these structures.
